# Adapting the Short Digital Stress Scale (SDSS) into Turkish: validation and psychometric evaluation in adults

**DOI:** 10.3389/fpsyg.2025.1701641

**Published:** 2026-01-12

**Authors:** Ali Geris, Erol Esen, Yağmur Soylu

**Affiliations:** 1 Department of Design Sciences, Lund University, Lund, Sweden; 2 Department of Computer Education and Instructional Technologies, Manisa Celal Bayar University, Manisa, Türkiye; 3 Department of Guidance and Psychological Counseling, Manisa Celal Bayar University, Manisa, Türkiye; 4 Department of Guidance and Psychological Counseling, Dokuz Eylul University, İzmir, Türkiye

**Keywords:** digital stress, Short Digital Stress Scale, psychometric properties, Turkish adaptation, mental health

## Abstract

**Introduction:**

This study aimed to adapt the Short Digital Stress Scale (SDSS) into Turkish and to evaluate its psychometric properties in an adult population. Digital stress has become increasingly relevant with the widespread and continuous use of digital technologies, highlighting the need for valid and reliable instruments across cultural contexts.

**Methods:**

A total of 276 adults (72.8% female, 27.2% male; M_age = 27.34, SD = 9.84) completed the SDSS together with established psychological measures, including the Bergen Social Media Addiction Scale, the Short-Form UCLA Loneliness Scale, the Liebowitz Social Anxiety Scale, and the Beck Depression Inventory-II. Confirmatory factor analysis was conducted to test the original five-factor structure of the SDSS. Convergent and concurrent validity were examined through composite reliability (CR), average variance extracted (AVE), and correlation analyses. Internal consistency was assessed using Cronbach's alpha.

**Results:**

Confirmatory factor analysis supported the original five-factor structure—availability stress, approval anxiety, fear of missing out, connection overload, and online vigilance—with good model fit (χ^2^/df = 1.32, GFI = 0.99, SRMR = 0.03, RMSEA = 0.03, AGFI = 0.98, CFI = 0.99). Factor loadings ranged from 0.36 to 0.93 (*p* < 0.001). CR and AVE values indicated convergent validity for all factors except connection overload. Digital stress showed significant positive correlations with social media addiction, loneliness, social anxiety, and depression. Internal consistency was acceptable (α = 0.77; subdimensions = 0.67–0.85).

**Discussion:**

The findings demonstrate that the Turkish version of the SDSS is a valid and reliable instrument for assessing digital stress among adults. The scale offers a concise and contextually appropriate tool for researchers and practitioners examining the psychological impact of digital engagement in Turkish society.

## 1 Introduction

In recent years, digital technologies have deeply permeated all aspects of daily life, reshaping how individuals interact, communicate, and access information. With the proliferation of smartphones, social media platforms, and online services, individuals are increasingly embedded in a socio-technical system that extends beyond physical boundaries. This transformation has fostered a global structure where socialization and communication are mediated through technology-driven platforms (Chayko, 2018). Digital tools such as mobile phones, tablets, and wearable devices have become extensions of personal identity, influencing daily routines and social presence (Gardner and Davis, 2013). The widespread and uninterrupted use of these devices has contributed to a continuous state of connectivity and psychological pressure to remain visible and responsive in digital spaces (We Are Social & Meltwater, 2023). As this constant engagement deepens, scholars have begun to examine its cognitive, emotional, and behavioral consequences within the framework of digital stress (Funke et al., 2025; Elavsky et al., 2022; Goswami and Deshmukh, 2023).

As digital technologies increasingly shape modern life, individuals are subjected to a new form of cultural normativity characterized by uninterrupted connectivity, real-time responsiveness, and perpetual visibility. Within this digital ecosystem, users encounter a constant flow of notifications, social updates, and interaction demands, which may lead to emotional fatigue and cognitive overload (Goswami and Deshmukh, 2023; Funke et al., 2025). The pressure to remain visible and active on social media platforms has been associated with heightened social comparison, fear of missing out, and the need for continuous validation through likes, comments, or follower counts (Gardner and Davis, 2013; Ünüvar, 2020). For many, smartphones function as psychological tethers, creating a dependency in which moments of disconnection can trigger anxiety, irritability, or restlessness (Körmendi et al., 2016; Kocabaş and Korucu, 2018). These psychosocial pressures, embedded within technology-mediated participation, contribute to attentional dispersion, emotional exhaustion, and behavioral dependency on digital interaction patterns (Chayko, 2018; Aksu and Işıklı, 2019).

While digital stress represents a broad psychological response to the cognitive, emotional, and behavioral demands of technology use, several related but distinct conditions have also emerged in the digital age. Among these, nomophobia defined as the fear of being without one's mobile phone has been identified as a prevalent phenomenon, particularly among younger individuals (Türen et al., 2017; King et al., 2013). Similarly, the Fear of Missing Out (FoMO), characterized by a persistent concern that others may be having rewarding experiences without one's involvement, often drives compulsive social media use and digital over-engagement (Yıldırım and Kişioğlu, 2018; Körmendi et al., 2016). The literature also highlights digital loneliness and digital fatigue as consequences of constant connectivity, whereby individuals report feelings of isolation despite extensive online activity (Goswami and Deshmukh, 2023; Ünüvar, 2020). In addition, modern digital life has given rise to more nuanced behavioral disruptions such as stalker-like monitoring on social platforms, compulsive digital hoarding, digital amnesia, and prolonged binge-watching (Akmeşe and Deniz, 2017; Jenner, 2017; van Bennekom et al., 2015; Wilmer et al., 2017). Although these conditions reflect the psychological toll of excessive digital exposure, they differ from digital stress in that they represent specific behavioral manifestations rather than a multifaceted stress response to technology-mediated demands (Ünüvar, 2020).

Building on these related but more specific digital-age conditions, researchers have increasingly conceptualized digital stress as a distinct and multifaceted construct arising from the cognitive, emotional, and behavioral demands of technology use. Digital stress is not solely a consequence of screen time but is shaped by a broader network of expectations, including constant availability, rapid responsiveness, and the need for ongoing social validation in online spaces (Funke et al., 2025; Goswami and Deshmukh, 2023). Individuals may feel overwhelmed by the volume of digital interactions, frequent multitasking across platforms, or the fear of social exclusion when disconnected, all of which contribute to emotional exhaustion and diminished well-being (Elavsky et al., 2022). Among adults, this burden is intensified by the simultaneous management of personal, professional, and social obligations within digital environments, leading to experiences such as connection overload, approval anxiety, and online vigilance (Hall et al., 2021; Funke et al., 2025). Together, these features illustrate a complex phenomenon in which digital participation while offering substantial opportunities also produces sustained psychological strain that warrants systematic measurement and intervention.

### 1.1 The Present study

Accurately measuring digital stress is crucial due to the growing integration of digital platforms into daily life. Researchers have developed several comprehensive tools to assess digital stress, but many instruments, such as the original 24-item Digital Stress Scale proposed by (Hall et al. 2021), present practical limitations in applied settings. Lengthy forms may cause participant fatigue, reduce response accuracy, and increase dropout rates, especially when researchers administer them alongside multiple psychological inventories (Funke et al., 2025). To address these challenges, researchers have increasingly emphasized the value of short-form instruments that preserve core construct validity while minimizing cognitive burden. (Funke et al. 2025) therefore introduced the 10-item Short Digital Stress Scale (SDSS) and demonstrated its strong psychometric properties and structural validity across samples from Germany, Italy, and Japan. Short forms also offer notable advantages for cross-cultural adaptation, as simplified translation procedures facilitate pilot testing and suit large-scale or rapid assessment contexts. This shift toward brief yet theoretically grounded measurement tools aligns with broader trends in psychological assessment that aim to balance empirical rigor with usability and participant engagement (Goswami and Deshmukh, 2023; Hall et al., 2021).

Despite the growing interest in digital stress as a psychological construct, culturally adapted and psychometrically validated tools for assessing digital stress among Turkish-speaking adults remain limited. Existing studies in Türkiye have predominantly focused on adolescents or generalized screen time behaviors, often overlooking the distinct emotional and cognitive demands experienced in adult digital life (Parlak et al., 2023; Aksu and Işıklı, 2019; Kocabaş and Korucu, 2018). Addressing this gap, the present study aimed to adapt the Short Digital Stress Scale (SDSS) developed by (Funke et al. 2025) into Turkish and examine its psychometric properties in an adult sample. In addition to evaluating the scale's construct validity through factor analyses, the study assessed concurrent validity by investigating associations between digital stress and psychological constructs known to correlate with digital stress, including loneliness, social media addiction, depression, and social anxiety (Goswami and Deshmukh, 2023; Funke et al., 2025). By focusing on adults and incorporating culturally grounded validation procedures, this study offers a reliable and contextually appropriate measurement tool that can support researchers, clinicians, and educators in understanding and addressing the psychological challenges associated with digital engagement in contemporary Turkish society.

## 2 Methodology

### 2.1 Participants

The convenience sampling method was employed to create the study group. Data were collected via an online survey instrument. The optimal participant number for scale adaptation studies is a topic of ongoing debate; however, prior research suggests a minimum of 200 participants (Comrey and Lee, 1992; Singh et al., 2016). Initially, 281 adults completed the online survey. However, following a data quality check, responses from five participants who incorrectly answered control items were excluded. As a result, the final sample comprised 276 participants. Among these, 201 (72.8%) identified as female and 75 (27.2%) as male. Participant ages ranged from 18 to 58 years, with a mean age of 27.34 (SD = 9.84). In terms of marital status, 65 participants (24.6%) were single, while 211 (76.4%) were married. Regarding education, 128 participants (46.4%) graduated from high school, while 148 (53.6%) held a university degree. Additionally, participants rated their socioeconomic status on a 10-point scale (1 = very low, 10 = very high), with a mean perceived socioeconomic status of 6.11 (SD = 1.74).

### 2.2 Procedures

The present study obtained ethical approval from the Social and Human Sciences Scientific Research and Publication Ethics Committee of Manisa Celal Bayar University (Meeting Date: December 23, 2024; Meeting No: 2024/17; Decision No: 19). Participation in the study was entirely voluntary, and both anonymity and confidentiality were strictly maintained throughout the research process. The participants received no form of financial compensation for their involvement, and data were collected using Google Forms, a platform secured by Google's robust security measures. The information collected was stored in an encrypted format on Google Drive, and no personally identifiable information was gathered, ensuring that all data remained anonymous. Additionally, access restrictions were implemented to ensure that only the researcher could view the data. When participants access the designated research link, they are presented with an informed consent page. This page provides detailed information about the researchers, the research purpose and methods, participants' rights, voluntariness, and confidentiality. Access to the online survey is granted only after participants have reviewed this information and confirmed their informed consent. To prevent data duplication during the collection process, the “Limit to one response” feature was enabled, requiring participants to log in with their Google accounts.

After obtaining the necessary permissions from the developers to adapt the MCFS into Turkish, the researchers followed the recommendations set forth by (Beaton et al. 2000). The initial translation of the scale into Turkish was carried out by two academics with expertise in counseling and English language teaching, in accordance with the guidelines that advocate for the involvement of independent translators from various academic disciplines. Following this, the author and the two translators collaborated to create a synthesis of the translations during a meeting, where they discussed and resolved any translation-related issues. A second English literature academic, who was not familiar with the original scale, conducted a back-translation of the items. Subsequently, two counseling experts verified the clarity and comprehensibility of all the items. The back-translated version was subsequently disseminated to the developers of the original scale, who conveyed their satisfaction with the translation. This led to the creation of the pilot application form.

The findings from the pilot study are particularly significant, as they provide insights into the comprehensibility of the items, the scale's response time, and any aspects that respondents found unclear (Cohen, 2005). The pilot study was conducted face-to-face with 15 adult volunteers. After this initial implementation phase, each participant was interviewed individually, and feedback was gathered regarding the clarity and comprehensibility of the items in the measurement tool. The final form for adapting the SDSS into Turkish was shaped based on the feedback indicating that the statements in the scale were clear and easily understood.

### 2.3 Materials

#### 2.3.1 Short digital stress scale (SDSS)

The SDSS is a ten-item scale developed by (Funke et al. 2025) to assess perceived digital stress in adults. Each item is rated on a seven-point Likert scale, ranging from “1 = never” to “7 = always”. Higher total scores reflect greater levels of digital stress. The scale is based on a five-dimensional construct, which includes availability stress, approval anxiety, fear of missing out, connection overload, and online vigilance, paralleling the 24-item version created by (Hall et al. 2021). The SDSS was initially validated in Germany, Italy, and Japan using three independent samples. Reported Cronbach's alpha coefficients were 0.90, 0.93, and 0.85, respectively. Confirmatory factor analysis (CFA) demonstrated strong model fit for the five-factor structure in each sample (Germany: χ^2^/df = 1.37, TLI = 0.99, CFI = 1.00, RMSEA = 0.04; Italy: χ^2^/df = 1.83, TLI = 0.98, CFI = 0.99, RMSEA = 0.06; Japan: χ^2^/df = 1.16, TLI = 1.00, CFI = 1.00, RMSEA = 0.03) (Funke et al., 2025). In the current study, the Cronbach's alpha coefficient for the Turkish version of the SDSS was calculated at 0.77, indicating good consistency.

#### 2.3.2 Bergen social media addiction scale (BSMAS)

The BSMAS is a unidimensional instrument developed to evaluate individuals' social media experiences over the past year (Andreassen et al., 2016). It has six items, each rated on a five-point Likert scale from “1 = very rarely” to “5 = very often”. Higher scores show a greater dependence on social media. In its original study, Cronbach's alpha was 0.88, and test-retest reliability was 0.83 (Andreassen et al., 2016). The tool was adapted into Turkish by (Demirci 2019). In the current study, the BSMAS had a Cronbach's alpha of 0.83, showing excellent internal consistency.

#### 2.3.3 The short-form UCLA Loneliness Scale (ULS-6)

The ULS-6 is a concise instrument developed to assess loneliness in adults. It was developed by (Neto 2014) as a shortened version of the 20-item UCLA Loneliness Scale. The ULS-6 consists of six items, each rated on a four-point Likert scale from 1 (never) to 4 (often), with higher scores reflecting greater loneliness. The original validation study reported strong internal consistency, with a Cronbach's alpha of 0.82. The Turkish adaptation, carried out by (Sarıçam 2023), who repoerted Cronbach's alpha was 0.77. In the present study, the Cronbach's alpha for the ULS-6 was calculated as 0.76, reflecting good reliability.

#### 2.3.4 Liebowitz social anxiety scale (LSAS)

The scale, developed by (Liebowitz 1987), assesses the severity of fear and avoidance in social situations. (Heimberg et al. 1999) examined its validity and reliability. The LSAS includes 24 items divided into two subscales: fear or anxiety and avoidance behavior. Each subscale uses a four-point Likert scale (0 = none/never to 4 = severe/usually). Higher LSAS scores indicate greater social anxiety symptom severity. The LSAS has shown adequate internal consistency, with Cronbach's alpha ranging from 0.81 to 0.92 (Heimberg et al., 1999). A Turkish validation was conducted by (Soykan et al. 2003), who reported a Cronbach's alpha of 0.98 and test-retest reliability of 0.97. In the present study, the Cronbach's alpha for the LSAS was calculated as 0.89, reflecting excellent internal consistency.

#### 2.3.5 Beck depression inventory-II (BDI-II)

The Beck Depression Inventory-II (BDI-II) is a 21-item self-report tool for assessing depressive symptom severity (Beck et al., 1996). Suitable for ages 13–80, each item uses a four-point Likert scale (0 = never to 3 = usually). The original study reported high internal consistency (Cronbach's alpha = 0.93) (Beck et al., 1996). (Kapci et al. 2008) adapted the BDI-II for Turkish, reporting internal consistency of 0.90 (nonclinical) and 0.89 (clinical) groups, with high test-retest reliability (r = 0.94). In this study, the BDI-II's Cronbach's alpha was 0.84, indicating excellent internal consistency.

#### 2.3.6 Demographic form

The demographic information form gathered data on participants' background characteristics. This form included questions about gender, age, marital status, level of education, and perceived socioeconomic status.

### 2.4 Data analysis

To enhance reliability in the online data collection process, control items intended to verify participant attentiveness were incorporated into the online survey. Prior to the main analyses, participants' responses to these control items were evaluated. Although data were collected from 281 participants, the responses of five participants who answered the control items incorrectly were excluded from the data set. Consequently, analyses were conducted with responses from 276 participants. While the optimal sample size for scale adaptation studies is still debated, previous research recommends a minimum of 200 participants (Comrey and Lee, 1992; Singh et al., 2016). Therefore, the sample size was deemed sufficient for the scale adaptation analyses.

Construct validity of the Turkish version of the SDSS was assessed through CFA using the maximum likelihood estimation method. Prior to performing the CFA, both univariate and multivariate normality assumptions were carefully evaluated. An analysis of the skewness and kurtosis values for all scales indicated that they fell within the acceptable range of -1.5 to +1.5, confirming univariate normality (Tabachnick et al., 2007). Additionally, multivariate normality was established through the elliptical distribution observed in the scatter plot matrix (Sheather, 2009).

To evaluate how well the proposed model represents the observed data, the assessment of model fit was conducted using several indices, including the chi-square to degrees of freedom ratio (χ^2^/df), the goodness of fit index (GFI), the standardized root mean square residual (SRMR), the root mean square error of approximation (RMSEA), the adjusted goodness of fit index (AGFI), and the comparative fit index (CFI). These indices were selected based on the methodologies outlined by (Kline 2005) and (Schermelleh-Engel et al. 2003). Similarly, the cut-off values suggested by (Kline 2005) and (Schermelleh-Engel et al. 2003) were used in the interpretation of fit indices. Furthermore, the convergent validity of the Turkish form of the SDSS was evaluated by calculating the composite reliability (CR) and average variance extracted (AVE) values.

The concurrent validity was assessed by calculating Pearson correlation coefficients among the SDSS, the BSMA, the UCL-6, the LSAS, and the BDI-II. The reliability of the tool was evaluated by employing Cronbach's alpha coefficient.

## 3 Findings

### 3.1 Construct validity of SDSS Turkish form

**Figure 1:**
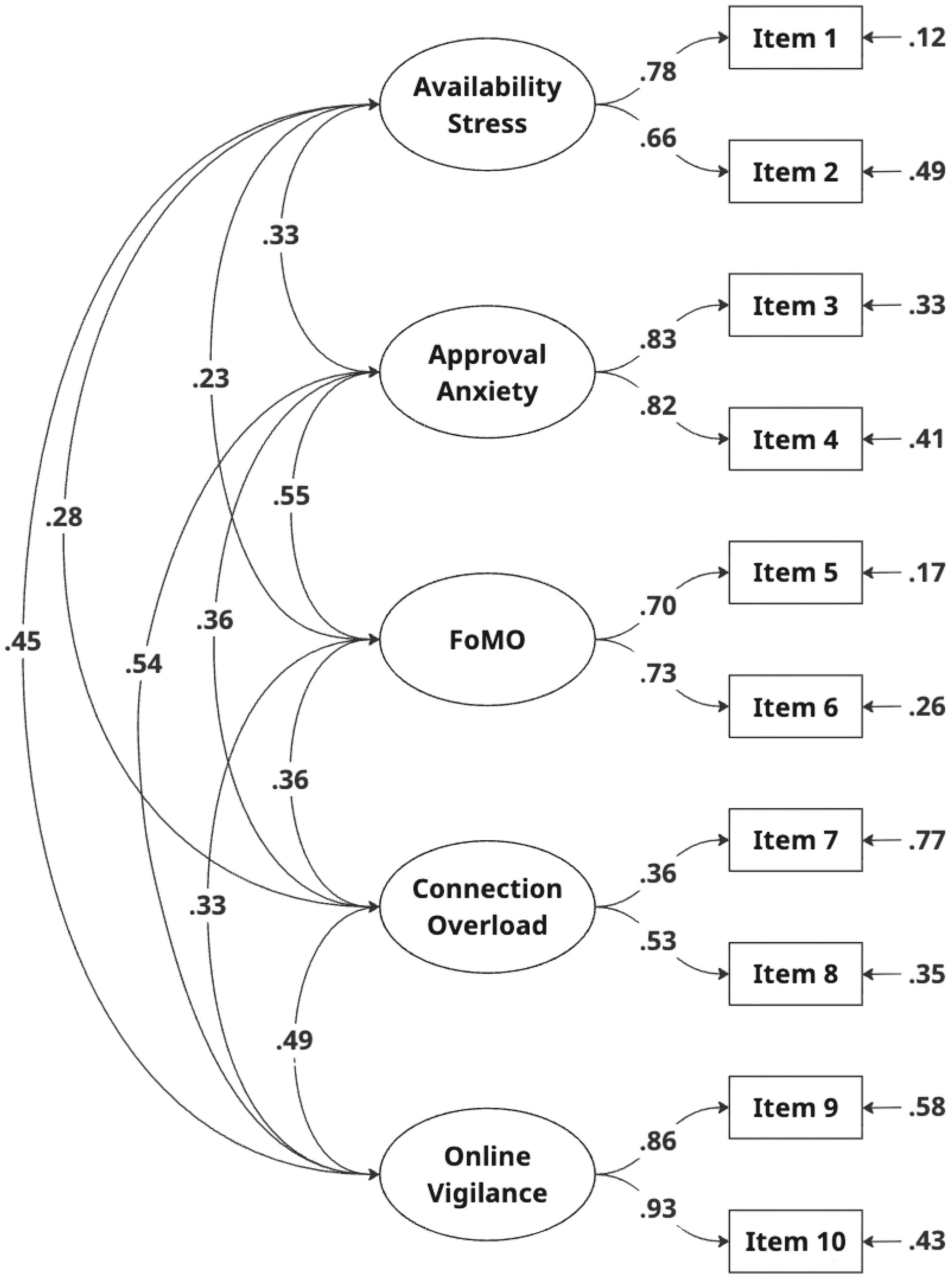
Path diagram of SDSS Turkish form.

The original SDSS is organized as a five-factor model. CFA using maximum likelihood estimation was conducted to assess the original five-factor structure in the Turkish context, as shown in Figure 1. The model demonstrated a good fit, as evidenced by the following fit indices: χ^2^(25) = 33.106, *p* < 0.001, χ^2^/df = 1.32, GFI = 0.99, SRMR = 0.03, RMSEA = 0.03, AGFI = 0.98, and CFI = 0.99. These values meet the “good fit” criteria established by (Schermelleh-Engel et al. 2003) and (Kline 2005) (see Table 1).

**Table 1:** Model fit indices for the SDSS Turkish form.

**Fit indices**	**Good fit**	**Acceptable fit**	**Measure**
χ^2^/df	0 ≤ χ^2^/df ≤ 2	2 < χ^2^/df ≤ 3	1.32
GFI	0.95 ≤ GFI ≤ 1.00	0.90 ≤ GFI < 0.95	0.99
SRMR	0 ≤ SRMR ≤ 0.05	0.05 < SRMR ≤ 0.10	0.03
RMSEA	0 ≤ RMSEA ≤ 0.05	0.05 < RMSEA ≤ 0.08	0.03
AGFI	0.90 ≤ AGFI < 1.00	0.85 ≤ AGFI < 0.90	0.98
CFI	0.97 ≤ CFI ≤ 1.00	0.95 ≤ CFI < 0.97	0.95

Cut-off values from (Kline 2005) and (Schermelleh-Engel et al. 2003) were used as references.

Furthermore, the results of the CFA indicated that the item loadings for the SDSS Turkish form ranged from 0.36 to 0.93, *p* < 0.001 (Table 2). The factor loadings surpassing the recommended threshold of 0.30 suggest that all items demonstrated a significant degree of saturation (Harrington, 2009; Kline, 2005).

**Table 2:** Factor loadings for SDSS Turkish form items.

**Factor**	**CR**	**AVE**	**Items**	**Factor Loadings**	***p***
Availability stress	0.74	0.59	Item 1	0.78	< 0.001
			Item 2	0.66	< 0.001
Approval anxiety	0.81	0.68	Item 3	0.83	< 0.001
			Item 4	0.82	< 0.001
Fear of missing out	0.68	0.51	Item 5	0.70	< 0.001
			Item 6	0.73	< 0.001
Connection overload	0.58	0.41	Item 7	0.36	< 0.001
			Item 8	0.53	< 0.001
Online vigilance	0.89	0.80	Item 9	0.86	< 0.001
			Item 10	0.93	< 0.001

This study investigated convergent validity as an aspect of construct validity. The Composite Reliability (CR) and Average Variance Extracted (AVE) values were computed for each factor of the scale (see Table 2). The CR and AVE values for the factors of availability stress, approval anxiety, fear of missing out, and online vigilance were found to exceed the critical thresholds of CR ≥ 0.60 and AVE ≥ 0.50, indicating that the SDSS Turkish Form demonstrates good convergent validity (Bagozzi and Yi, 2012; Hair et al., 2006). Conversely, the CR (0.58) and AVE (0.41) values for the connection overload factor remained below these critical thresholds. Some researchers suggest that AVE values above 0.40 are acceptable, provided that CR is adequate (Fornell and Larcker, 1981; Lam, 2012).

### 3.2 Concurrent validity of the SDSS Turkish form

The concurrent validity of the Turkish version was assessed through Pearson correlation analyses involving the SDSS, BSMA, UCL-6, LSAS, and BDI-II scores of the participants (see Table 3). The results indicated a moderate positive correlation between the SDSS and both the BSMA and LSAS scores (r = 0.51, *p* < 0.01; r = 0.46, *p* < 0.01, respectively). Additionally, a weak positive correlation was found between the SDSS and UCL-6 (r = 0.26, *p* < 0.01), as well as between BDI-II scores (r = 0.20, *p* < 0.01) (Salkind, 2016). Based on predictions derived from established theoretical frameworks, these findings suggest a positive relationship between digital stress levels and social media addiction, loneliness, social anxiety, and depressive symptoms.

**Table 3:** Correlation coefficients for SDSS, BSMA, UCL-6, LSAS and BDI-II.

	**1**	**2**	**3**	**4**	**5**
1. SDSS - Digital stress	-				
2. BSMA - Social media addiction	0.51^**^	-			
3. UCL-6 - Loneliness	0.26^**^	0.34^**^	-		
4. LSAS - Social anxiety	0.46^**^	0.39^**^	0.48^**^	-	
5. BDI-II - Depression	0.20^**^	0.35^**^	0.40^**^	0.35^**^	-

^*^p < 0.05, ^**^p < 0.01.

### 3.3 Reliability of the SDSS Turkish form

The reliability of the measurement tool was assessed by calculating the Cronbach's alpha coefficient for internal consistency. The overall Cronbach's alpha value for the entire scale was found to be 0.77. Additionally, the coefficients for the subdimensions, availability stress, approval anxiety, fear of missing out, connection overload and online vigilance were determined to be 0.83, 0.83, 0.85, 0.67, and 0.80, respectively.

## 4 Discussion

Due to the increasing use of digital technologies, it is considered important to study the concept of digital stress, which is a psychological construct related to the use of digital technologies but distinct from behaviors such as screen time, in the adult population. Based on this, the aim of this study was to adapt the Short Digital Stress Scale (SDSS) developed by (Funke et al. 2025) into Turkish and evaluate its psychometric properties in an adult sample. The Turkish adaptation of the Short Digital Stress Scale (SDSS) was carried out, and its psychometric properties were rigorously assessed in an adult sample ranging in age from 18–58 years. The original five-dimensional factor structure of the SDSS, consisting of 10 items, was tested, and its criterion-related validity and reliability levels were examined. Model fit indices (χ^2^/df, GFI, SRMR, RMSEA, AGFI, CFI) indicated that the SDSS has a statistically acceptable psychometric structure. The significant correlations identified between the SDSS and the BSMAS, UCL-6, LSAS, and BDI-II demonstrated that the SDSS has criterion-related validity. Cronbach's alpha coefficients calculated for the items in the SDSS indicate that the target measurement tool is reliable. In conclusion, the findings of this study demonstrate the validity and reliability of the Turkish adaptation of the SDSS as a measurement instrument for assessing digital stress levels in adults.

The findings obtained in the present study appear to be consistent with those reported in the original scale study on SDSS by (Funke et al. 2025). As in the original study, the five-dimensional and 10-item original structure, which includes the factors of availability stress, approval anxiety, fear of missing out, connection overload, and online vigilance, was confirmed in the target sample. Consistent with the findings of the original study, it was observed in the present study that all scale items significantly represented their respective latent constructs. In this study, factor loadings were found to be 0.36 and above. All standardized factor loadings were found to be significant and within acceptable ranges (Nunnally, 1978). Furthermore, when model fit was evaluated through confirmatory factor analysis, these values aligned with the "good fit" criteria put forth by (Schermelleh-Engel et al. 2003) and (Kline 2005).

Although the five-factor structure of the SDSS was supported, the convergent validity indicators of the connection overload dimension were comparatively weaker. This outcome is not unexpected for a factor represented by only two items, as both AVE and CR are known to be highly sensitive to item count, and lower inter-item correlations may naturally reduce these coefficients (DeVellis and Thorpe, 2021; Netemeyer et al., 2003). Moreover, reliability indicators such as Cronbach's alpha while ideally 0.70 or above are considered acceptable at 0.60 or higher in short scales, particularly when model fit indices demonstrate strong overall adequacy, as observed in this study. The relatively modest loading of Item 7 further suggests that subtle linguistic or interpretive differences may influence responses. Cross-cultural patterns reported in the original SDSS validation (Funke et al., 2025) similarly showed minor variability in this subdimension, supporting the notion that perceptions of digital overload depend on culturally shaped norms of multitasking and constant availability (Hall et al., 2021). Additionally, age-related differences between samples may partially explain item-level variation: while the original study included older participants (mean ages 38–54 across countries), the Turkish sample consisted predominantly of young adults (M = 27), who tend to view continuous connectivity as normative rather than overwhelming (Ünüvar, 2020; Goswami and Deshmukh, 2023). Considering the combined evidence robust model fit indices, acceptable reliability levels, and theoretically grounded explanations for item-level variation the connection overload factor can still be interpreted as psychometrically adequate, although future studies should reassess this subdimension in more heterogeneous samples.

In the present study, significantly positive correlations were found between the total scores obtained from digital stress levels and social media addiction, loneliness, social anxiety, and depressive symptoms. In other words, empirical findings supported the concurrent validity of SDSS in the target sample. Taken together, these results validate the SDSS while also revealing a differentiated pattern of associations that warrants further conceptual consideration. The weaker correlations with depressive symptoms and loneliness, in particular, suggest that digital stress operates within a complex behavioral ecology shaped by individuals' engagement with digital environments. This complexity becomes more intelligible when situating digital stress within contemporary usage patterns of digital tools. Indeed, Balcı and Kaya (2023) show in their study with 390 university students that participants actively use digital tools to cope with stressful situations and avoid negative feelings. This finding also explains the relatively weak correlation between digital stress and depression (r = 0.20) and loneliness (r = 0.26). Additionally, international research also emphasizes that young people engage more with digital tools (Chaudron, 2015; Media, 2017). While Cronbach's alpha coefficients for the subdimensions of SDSS were reported to be 0.88 and above in the original study, in the present study, internal consistency coefficients ranged between 0.67 and 0.85. Although values of 0.70 and above are generally accepted as a benchmark for internal consistency in the literature, it has recently been observed that researchers also consider values of 0.60 and above to be within acceptable limits (Jain and Angural 2017). In this context, the findings obtained in the present study suggest that SDSS is a reliable measurement tool. In this study, the total internal consistency coefficient of SDSS was calculated as 0.77. Based on this finding, it can be seen that the overall reliability level is above the accepted threshold.

## 5 Practical implications

Due to the increasing frequency and variety of digital tool usage (Chaudron, 2015; Media, 2017), research on the psychological effects of digital tool usage has also begun to attract more attention. In this context, the Digital Stress Scale for adults is considered to be useful not only for researchers interested in the concept of digital stress but also for professionals working in the field of mental health.

Today, with digitalization, digital stress is considered an area of stress that needs to be addressed (Özyılmaz, 2021). Measuring adults' levels of digital stress will facilitate the shaping of necessary intervention approaches by examining experiences related to digital stress in the field of application, in addition to determining digital stress levels. When evaluating the psychological effects of digital stress, its relationship with psychological well-being is noteworthy (Khetawat and Steele, 2023). At this point, mental health practitioners bear significant responsibility in addressing these emerging dynamics through informed assessment and therapeutic strategies.

## 6 Limitations and future research

Despite its contributions, the study has certain methodological and contextual limitations that warrant consideration. The findings obtained in this research are limited to the responses of 281 individuals aged between 18 and 58 who answered the measurement tool in an online context. The limited sample of adults accessible through online platforms represents a notable limitation regarding the generalizability of the findings. A further limitation of utilizing a convenience sample is the presence of demographic imbalances. Although the participant age range spans from 18 to 58 years, the mean age of 27.34 indicates a predominance of young adults. Additionally, the sample consists primarily of female participants. These demographic imbalances are particularly important because age and gender may influence responses to scale items and underlying constructs (Stuart et al., 2015). A sample skewed toward younger, predominantly female participants may restrict the generalizability of the findings. To enhance the generalizability of the findings, future research should strive for more balanced age and gender representation and assess measurement invariance across demographic groups.

In addition to these limitations, another consideration concerns the study's measurement tool. The relatively weaker convergent validity indicators for the connection overload factor warrant cautious interpretation. Future research should re-examine this dimension using more diverse samples to determine whether the observed pattern reflects cultural or sample-specific characteristics. Notwithstanding its limitations, the current research addresses a critical void in the existing national literature. The Turkish adaptation of the SDSS carried out in this study is expected to pave the way for further research into adults' digital stress levels within the national context. It can also be argued that the adaptation of the 10-item short form of the SDSS in the present study provides a brief, economical, and practical measurement tool for researchers conducting multivariate and longitudinal studies. Given the pervasive integration of digital technologies into daily life, digital stress is likely to emerge as a central topic within the domains of counseling and psychology.

In future studies, examining adults' digital stress levels in relation to various variables (such as age, gender, occupation, family structure, etc.) and identifying factors that influence digital stress may further develop the intervention areas for mental health professionals. As a psychological construct that affects adults in both work and family environments, digital stress may offer a new intervention area for mental health professionals in supporting individuals' quality of life, well-being, and happiness. Today, adults, just like children and adolescents, are increasingly spending more time in virtual environments rather than engaging in face-to-face, natural interactions, relationships, and experiences, which may weaken the healthy development of coping mechanisms. In this context, it is considered important to investigate the concept of digital stress in studies involving adults.

In conclusion, considering the widespread use of digital technologies that affect all areas of daily life (Chayko, 2018), digital stress emerges as a significant psychological construct. Although digital stress has mostly been addressed in studies conducted with adolescents (Nick et al., 2022; Steele et al., 2020; Winstone et al., 2023), it has emerged as a variable that needs to be examined across different developmental periods as well. Furthermore, a measurement tool designed to assess digital stress levels in adults. In addition to focusing on children, adolescents, or screen time behaviors may open the door to new studies that focus on both adults and the cognitive and emotional aspects of digital technology use. The findings obtained in such studies may contribute to shaping intervention programs aimed at reducing digital stress. It can be said that this tool may serve as a guide for offering research and practical interventions to support adults in managing the psychological aspects of digital technology use.

## Data Availability

The original contributions presented in the study are included in the article/supplementary material, further inquiries can be directed to the corresponding author.
